# Consecutive Use of the 52 mg Levonorgestrel-releasing Intrauterine System: Variations in Bleeding Patterns

**DOI:** 10.1055/s-0040-1708092

**Published:** 2020-04

**Authors:** Barbara Zantut Wittmann, Ilza Monteiro, Cássia Juliato, Arlete Fernandes

**Affiliations:** 1 Department of Obstetrics and Gynecology, Universidade Estadual de Campinas, Campinas, SP, Brazil

**Keywords:** levonorgestrel intrauterine system, consecutive use, bleeding patterns, Sistema Intrauterino De Levonorgestrel, uso consecutivo, padrão de sangramento

## Abstract

**Objective** Changes in bleeding patterns could influence the decisions of healthcare professionals to change the levonorgestrel-releasing intrauterine system (LNG-IUS) before 7 years of use, the recommended period of extended use. We evaluated changes in the bleeding patterns of users of the 52 mg LNG-IUS at the end of use of the first (IUS-1) and during the second device (IUS-2) use.

**Methods** We performed an audit of the medical records of all women who used two consecutive LNG-IUSs at the Family Planning clinic. We evaluated the sociodemographic/gynecological variables, the length of use, and the bleeding patterns reported in the reference periods of 90 days before removal of the IUS-1 and at the last return in use of IUS-2. We used the McNemar test to compare bleeding patterns. Statistical significance was established at *p* < 0.05.

**Results** We evaluated 301 women aged (mean ± SD) 32 (±6.1) years, with lengths of use of 68.9 (±16.8) and 20.3 (±16.7) months for the IUS-1 and IUS-2, respectively. No pregnancies were reported. Bleeding patterns varied significantly among women who used the IUS-2 for ≥ 7 months to 6 years when compared the bleeding patterns reported in IUS-1 use. Eighty-nine out of 221 (40%) women maintained amenorrhea and infrequent bleeding; 66 (30%) evolved to bleeding patterns with light flow, and 66 (30%) maintained or evolved to heavy flow patterns (*p* = 0.012). No differences were observed among the 80 women with ≤ 6 months of use.

**Conclusion** Changes in bleeding patterns occur during the use of LNG-IUS and should not be decisive for the early replacement of the device.

## Resumo

**Objetivo** Variações no padrão de sangramento podem afetar a decisão de troca do sistema intrauterino de levonorgestrel (SIU-LNG) antes do período de uso estendido recomendado de 7 anos. Nós avaliamos mudanças no padrão de sangramento de usuárias ao final do uso do primeiro SIU-LNG 52 mg (SIU-1) e durante o uso do segundo dispositivo (SIU-2).

**Métodos** Revisamos os prontuários de todas as mulheres que inseriram consecutivamente o SIU-LNG no ambulatório de Planejamento Familiar. Foram avaliadas as variáveis sociodemográficas/ginecológicas, o tempo de uso, e os padrões de sangramento relatados nos períodos de referência de 90 dias antes da remoção do SIU-1 e no último retorno em uso do SIU-2. Usamos o teste de McNemar para comparar os padrões de sangramento. A significância estatística foi estabelecida em *p* < 0,05.

**Resultados** Analisamos os dados de 301 mulheres com idade (média ± desvio padrão [DP]) de 32 (±6,1) anos e tempo de uso de 68,9 (±16,8) e 20,3 (±16,7) meses para o SIU-1 e SIU-2, respectivamente. Nenhuma gravidez foi relatada. Os padrões de sangramento variaram significativamente durante o uso do SIU-2 (≥ 7 meses a 6 anos) em relação ao padrão relatado no SIU-1. Oitenta e nove das 221 (40%) mulheres mantiveram amenorreia e sangramento infrequente; 66 (30%) evoluíram para padrões de sangramento com fluxo leve e 66 (30%) mantiveram ou evoluíram para padrões de fluxo intenso (*p* = 0,012). Não foram observadas diferenças entre as 80 mulheres que utilizavam o SIU-2 há ≤ 6 meses.

**Conclusão** Mudanças nos padrões de sangramento ocorrem durante o uso do LNG-IUS e não devem ser decisivas para a troca precoce do dispositivo.

## Introduction

The 52 mg levonorgestrel-releasing intrauterine system (LNG-IUS) is a long-acting, reversible contraceptive method, also used as treatment for heavy menstrual bleeding.^1^ ^2^ It has been widely used with high satisfaction rates, and many women opt for the insertion of a new IUS after the end of the approved lifespan of 5 years.^3^ ^4^ In addition, it has been reported that this device could be used beyond the approved 5-year lifetime, and data have been published up to 7 years of use, which improves cost-effectiveness.^5^ ^6^ ^7^ However, some users and healthcare professionals (HCPs) associate changes in bleeding patterns at the end of the approved lifespan with decreasing contraceptive efficacy of the method, which does not seem supported by the medical literature.^5^ ^6^ ^7^

The high effectiveness of LNG-IUS is attributable to two mechanisms of local action: the antiproliferative effect upon the endometrium, which induces amenorrhea, and the effect on cervical mucus, which impairs sperm penetration.^8^ ^9^ ^10^ The 52 mg LNG-IUS releases 20 µg/day immediately after device placement and declines over time to 10 to 12 µg/day up to 5 years of use; however, LNG has been detected in the 8^th^ year of use.^11^ ^12^ The serum mean (±SEM) LNG levels decreased from 253 ± 27 pg/ml (range, 86–760) during the first 2 months after placement to 137 ± 12 (range, 23–393) at 7 years of use and 119 ± 9 pg/ml (range, 110–129) at 8 years after placement.^11^ The reduction of LNG levels correlated to increments of endometrial thickness (from 2.8 ± -0.1 mm at 84 months of use to 3.8 ± -0.5 mm at 102 months of use).^11^ The authors reported that as LNG decreased over time, the amenorrhea rate decreased from 41.8% at 84 months to 31.5% at 102 months of use, but no correlations were found between serum LNG levels and bleeding patterns.^11^

Despite the lower release of LNG with over time use, the described contraceptive failure is 0.2/100 women-years (WY) and is similar to the rates reported for new LNG-IUSs loaded with 19.5 mg (Kyleena— Bayer PLC, Reading, Berkshire, UK), which release 16 µg/day and 7.4 µg/day of LNG at the 1^st^ and 5^th^ year of use, respectively, and only slightly less (0.4/100 WY) for the LNG-IUS loaded with 13.5 mg (Jaydess/Skyla – Bayer Canada, Toronto, ON, Canada), which releases 12 µg/day and 5 µg/day at the end of the 1^st^ and 3^rd^ year of use, respectively.^13^ ^14^ ^15^

Despite the large body of evidence concerning efficacy and bleeding patterns among 52 mg LNG-IUS users, information about the use of 2^nd^ or 3^rd^ consecutive LNG-IUSs is scarce.^1^ ^3^ ^4^ ^13^ ^16^ ^17^ It is not well established whether users of the LNG-IUS maintain the same bleeding patterns observed at the end of the 5-year approved lifespan after changing the device out for a new one or if the bleeding patterns change to a lighter or heavier flow. It is important to know if changes in the bleeding pattern still occur during the predicted period of high contraceptive efficacy of the method. This information may help to dispel the idea that the changes in menstrual pattern that take place in year 5 of using the method correspond to a decrease in the contraceptive efficacy.

The objective of our study was to assess and compare self-reported bleeding patterns of LNG-IUS users with reference periods of 90 days before the removal of first IUS (IUS-1), after the approved lifespan and same-day replacement with a second IUS (IUS-2), and at the last annual return visit of IUS-2.

## Methods

This was a retrospective cohort study conducted at the Family Planning clinic, Department of Obstetrics and Gynecology, The ethical committee of the institution approved the study protocol and authorized the data collection and analysis; the information was unidentifiable following collection. The medical records of all women who received a 52 mg LNG-IUS (Mirena—Bayer Oy, Turku, Finland) for contraception at our service and consecutively received an IUS-2 were included in the study. The data were collected from information contained in the medical records of the service. Women who did not have information about bleeding patterns in the medical records were not included. We obtained sociodemographic and obstetrics information, total length of use, bleeding patterns and rates and reasons for discontinuation of the IUS-2. The professionals of our service followed an interview script about the women's menstrual history at the time of insertion of the IUS, and the women are questioned about the menstrual bleeding presented in the last 90 days on subsequent return visits. They were categorized into 5 patterns: amenorrhea (no bleeding), infrequent bleeding (1–2 episodes of bleeding and/or spotting), frequent bleeding (> 5 episodes of bleeding and/or spotting), regular bleeding (3–5 episodes of bleeding and/or spotting), and prolonged bleeding (> 14 consecutive days of bleeding and spotting).^18^ For this study, we collected information on bleeding patterns self-reported by women in a reference period of the last 90 days before IUS-1 removal and 90 days before the last return visit to the clinic when using IUS-2. The patterns of “frequent” and “prolonged” bleeding were computed together for analysis as “frequent/prolonged” bleeding due to the scarce number of women with these patterns.

### Statistical Analysis

We used the χ^2^, Fisher exact, Mann-Whitney, and Kruskal-Wallis tests followed by a post-hoc Dunn test to identify the variables with association. For comparisons between bleeding patterns, we used the McNemar test. Statistical significance level was established at *p* < 0.05. The Statistical Analysis System (SAS) software program, version 9.4 (SAS Institute Inc., Cary, NC, USA) was used for the analysis.

## Results

Between 2007 and 2017, 12,570 LNG-IUSs were inserted at our service. We reviewed the medical charts of 316 women who received a 2^nd^ consecutive LNG-IUS on the same day as the removal of the 1^st^ one at the end of the approved lifespan. From those women, 15 were excluded due to lack of reliable information; consequently, we report information regarding 301 women with information about bleeding patterns. The mean (±standard error of the mean [SEM]) age at IUS-1 and IUS-2 insertion was 32.0 (±0.35) and 37.7 (±0.37), respectively. The length of use of IUS-1 and IUS-2 was 68.9 (±0.97) and 20.3 (±0.96) months, respectively. Most of the women attended ≤ 8 years of schooling and were living with a partner (Table 1).

**Table 1:** Characteristics of users of a second consecutive levonogestrel-releasing intrauterine system

Variables	n = 301 (100%) n (%)
Age at IUS-1 placement, mean (SEM)	32.0 (0.35)
Age at IUS-2 placement, mean (SEM)*	37.7 (0.37)
Schooling (years), n (%)**	
≤ 8	243 (82.6)
> 8	51 (17.1)
Marital status, n (%)***	
With a partner	246 (82.8)
Without a partner	51 (17.1)
Number of pregnancies, mean (SD)	1.7 (0.9)
Number of deliveries, mean (SD)	1.5 (0.8)
Length of use of the IUS-1 (months), mean (SEM)	68.9 (0.97)
Length of use of the IUS-2 (months), mean (SEM)	20.3 (0.96)

Abbreviations: IUS, intrauterine system; SD, standard deviation; SEM, standard error of the mean.

*Missing = 2 (299 women evaluated); **Missing = 7 (294 women evaluated); ***Missing = 4 (297 women evaluated).

We also observed that at the end of use of the IUS-1, 43.8%, 23.9%, 24.9%, and 7.3% of women had reported amenorrhea, infrequent, regular, and frequent/prolonged bleeding, respectively (Table 2). The length of use of the IUS-1 was different according to the bleeding pattern and was longer among women with amenorrhea compared with those with regular or frequent/prolonged bleeding; it was also longer among women with infrequent compared with those with frequent/prolonged bleeding (Table 2). Additionally, the length of use (IUS-1 + IUS-2) was significantly higher among women with amenorrhea compared with women with frequent/prolonged bleeding or regular bleeding (Table 2).

**Table 2:** Bleeding patterns reported at the end of use of the first intrauterine system and its relationship with some variables

Variables	Amenorrhea n = 132 (43.8%) n (%)	Infrequent n = 72 (23.9%) n (%)	Regular n = 75 (24.9%) n (%)	Frequent/prolonged n = 22 (7.3%) n (%)	*p-*value*
Age at IUS-1, mean (SD)¶	31.8 (6.1)	32.3 (6.0)	32.0 (6.0)	33.1 (7.7)	0.742
Age at IUS-2, mean (SD)^¥^	37.9 (6.2)	37.8 (6.4)*	37.1 (6.6)**	38.3 (8.4)	0.758
Number of pregnancies, mean (SD)	1.7 (0.9)	1.9 (0.8)	1.8 (0.9)	1.6 (1.4)	0.082
Number of deliveries, mean (SD)	1.5 (0.7)	1.7 (0.7)	1.6 (0.8)	1.4 (0.9)	0.183
Number of abortions, mean (SD)	0.1 (0.5)	0.1 (0.4)	0.3 (0.7)	0.1 (0.6)	0.362
Length of use of IUS-1, months, mean (SD)	73.0 (10.4)	68.7 (17.4)	63.8 (22.4)	63.1 (18.3)	0.0002^#^
Length of use of IUS-2, months, mean (SD)	21.9 (16.8)	16.6 (15.6)	20.0 (18.2)	23.5 (13.4)	0.055
Total length of use months, mean (SD)	95.4 (21.0)	85.4 (18.2)	84.3 (21.7)	86.5 (23.6)	0.0006^&^

Abbreviation: IUS-1, first intrauterine system; ISU-2, second intrauterine system; SD, standard deviation.

¶ Age at first placement; ^¥^Age at second placement; *Kruskal Wallis test; ^#^Post-hoc Dunn test – amenorrhea > frequent/prolonged and amenorrhea > regular; spotting > frequent/prolonged; ^&^Post-hoc Dunn test – amenorrhea > frequent/prolonged and amenorrhea > regular; *Missing = 1 (71 women evaluated); **Missing = 1 (74 women evaluated).

At the last visit using the IUS-2, we observed an increment of users with amenorrhea (50.8%) and infrequent bleeding (28.5%), and a low proportion of women with regular (15.6%) or frequent/prolonged bleeding (4.9%) (Table 3).

**Table 3:** Distribution of women according to bleeding patterns reported during the last 90 days of use of the 1^st^ intrauterine system and 90 days before the last return visit using the 2^nd^ intrauterine system

Bleeding pattern	Last 90 days of use of the IUS-1 n = 301 n (%)	Length of use of the IUS-2	
		≤ 6 months n = 80 n (%)	> 7 months–6 years n = 221 n (%)
Amenorrhea	132 (43.8)	35 (43.7)	118 (53.4)
Infrequent	72 (23.9)	24 (30.0)	62 (28.0)
Regular	75 (24.9)	17 (21.2)	30 (13.5)
Frequent/prolonged	22 (7.3)	4 (5.0)	11 (4.9)

Abbreviation: IUS, intrauterine system.

Table 4 shows the comparison between the frequencies of bleeding patterns presented at the end of use of the IUS-1 and at the last visit using the IUS-2. Of the 221 women who used the IUS-2 from 7 months to 6 years, only 89/221 women (40%) maintained the same patterns of amenorrhea and infrequent bleeding; 66 (30%) evolved to bleeding patterns with lighter flow, and 66 (30%) maintained or evolved to patterns with heavier flow than presented when in use of IUS-1 (*p* = 0.012). Among the 104 women with amenorrhea at the end of IUS-1 use, only 70 continued to experience amenorrhea (Table 4).

**Table 4:** Bleeding pattern variations during the last 90 days before removal of the 1^st^ intrauterine system and 90 days before last visit using the 2^nd^ intrauterine system for > 7 months to 6 years

Bleeding pattern with IUS-1	Bleeding pattern with IUS-2				
	Amenorrhea n = 118 n (%)	Infrequent n = 62 n (%)	Regular n = 30 n (%)	Frequent/prolonged n = 11 n (%)	*p-*value@
Amenorrhea, *n* = 104	70 (59.3)	25 (40.3)	3 (10.0)	6 (54.5)	0.012
Infrequent, *n* = 48	23 (19.5)	19 (30.6)	5 (16.6)	1 (9.0)	
Regular, *n* = 50	16 (13.5)	14 (22.5)	18 (60.0)	2 (18.1)	
Frequent/prolonged, *n* = 19	9 (7.6)	4 (6.4)	4 (13.3)	2 (18.1)	

@ McNemar test. Excludes the 80 women with length of use of the IUS-2 ≤ 6 months.

Of the 80 women who used the IUS-2 for only 6 months, there were no differences when comparing the bleeding patterns with those observed at the end of use of the IUS-1 (*p* = 0.1163). At the end of the data collection (September/2017), 277 (92%) women were still using the device and 9 were using a 3^rd^ LNG-IUS after the end of the approved lifespan. Also, 24 women had the device removed: 3 wished to become pregnant, 9 reached menopause, and 12 had expulsions.

## Discussion

Our results showed that women using a 52 mg LNG-IUS had significantly varied bleeding patterns over time while using this method. We emphasize that women with amenorrhea at the end of use of the IUS-1 also changed to other bleeding patterns like infrequent (24%), and even regular and frequent/prolonged bleeding (8.6%), during the use of the IUS-2.

Our findings are important to HCPs to determine how to counsel women on when to change the LNG-IUS and to consider the possibility of extended use for 7 years and beyond.^5^ ^7^ In our service, some HCPs have expressed concerns of reduced contraceptive effectiveness when women keep the same device beyond the manufacturer-recommended 5 years, mainly among women who reported changes in their bleeding patterns and especially if they experienced amenorrhea at any time during use and changed to other bleeding patterns after 5 years of use. The behavior of the HCPs for early removal and replacement of the IUS was reflected in the results presented in this report showing long periods of use for women with amenorrhea and short periods among women with other bleeding patterns.

Because no correlation was reported between serum LNG concentrations and bleeding patterns, we can speculate that low doses of LNG after the 5^th^ year of use of the 52 mg LNG-IUS maintain the high contraceptive efficacy independently of the observed bleeding patterns, which were observed previously.^6^ ^7^ ^11^ ^12^ ^19^

We found at end use of IUS-1 that amenorrhea was observed in 43.8% of users; this result is in agreement with a recent prospective study that described 41.8% of amenorrhea at 5 years of use of the LNG-IUS.^13^

Our results showed that women with amenorrhea displayed infrequent and regular bleeding patterns in the 2 study periods within the range described in previous prospective studies with slight variations, possibly due to the retrospective characteristic of our study.^4^ ^16^ ^17^ A Sweden-based study followed 82 women who underwent a 2^nd^ consecutive IUS placement after 7 years of use with the 1^st^ one, and the authors reported that 26% and 60% of participants experienced amenorrhea in the 1^st^ period for the 1^st^ IUS and for the last 5 years of use for the 2^nd^ device, respectively, while showing 70% and 28% with regular/scanty bleeding patterns while using the 1^st^ and 2^nd^ IUSs, respectively.^4^

In addition, a multicenter study of 204 women with a 15-month follow-up evaluated bleeding patterns during the 1^st^ LNG-IUS (between 4.3 and 4.9 years) and showed a mean of 7 and 8 days of bleeding/spotting at 90 days before and after changing the device, respectively, decreasing to 4 days after 1 year of use with the 2^nd^ IUS.^16^ Of the 204 women in that study, 170 women remained at follow-up, and the bleeding patterns described in the last 90 days 2 to 5 years after placement of the 2^nd^ IUS showed reductions of bleeding in 70% of the women, while > 49% of women experienced amenorrhea, which was associated with high rates of satisfaction and continuation.^17^ The results of our study were similar to those found in these prospective studies.^4^ ^16^ ^17^

The main limitation of the present study was the retrospective design, which could introduce bias. On the other hand, the main strength is that we evaluated the same cohort of women at two different moments, which may have contributed, at least in part, to the quality of the evaluation of the variations between bleeding patterns. Additionally, another strength is that we assessed the bleeding patterns 90 days before the last annual consultation of the IUS-2, which occurred between 7 months and 6 years after placement.

Our study has two major implications for HCPs who provide care in family planning. First, the bleeding patterns observed in LNG-IUS users did not change in the first 6 months after changing the device for a new one. Second, in the period from 7 months up to 6 years after the 2^nd^ device placement, it is expected that the bleeding patterns will vary from the pattern observed when removing the 1^st^ IUS. This information may help users and HCPs to more readily accept the extended use of the method and prevent both from interpreting changes in bleeding patterns as decreasing the efficacy of the method.

Although users of a second LNG-IUS reported high rates of amenorrhea and infrequent bleeding and lower rates of regular, frequent/prolonged bleeding after changing the LNG-IUS, some women also experienced bleeding pattern changes from light flow to frequent/prolonged flow, which has not been found to be linked to contraceptive efficacy.

As we evaluated the time of IUS-2 use for up to 6 years of use, a period in which its efficacy is still high, these changes in bleeding pattern did not appear to indicate a decrease in the contraceptive efficacy of the method. In addition, we did not have any pregnancy in the period of IUS-2 use.

## Conclusion

Changes in bleeding patterns occur during the use of LNG-IUS and should not be decisive for the early replacement of the device.

## References

[1] Hubacher D The levonorgestrel intrauterine system: reasons to expand access to the public sector of Africa Glob Health Sci Pract 2015 3 04 532 537. Doi: 10.9745/GHSP-D-15-00178 26681701 10.9745/GHSP-D-15-00178 PMC4682579

[2] Bahamondes L Valeria Bahamondes M Shulman L P Non-contraceptive benefits of hormonal and intrauterine reversible contraceptive methods Hum Reprod Update 2015 21 05 640 651. Doi: 10.1093/humupd/dmv023 26037216 10.1093/humupd/dmv023

[3] Backman T Huhtala S Luoto R Tuominen J Rauramo I Koskenvuo M Advance information improves user satisfaction with the levonorgestrel intrauterine system Obstet Gynecol 2002 99 04 608 613. Doi: 10.1016/s0029-7844(01)01764-1 12039121 10.1016/s0029-7844(01)01764-1

[4] Rönnerdag M Odlind V Health effects of long-term use of the intrauterine levonorgestrel-releasing system. A follow-up study over 12 years of continuous use Acta Obstet Gynecol Scand 1999 78 08 716 721. Doi: 10.1034/j.1600-0412.1999.780810.x 10468065

[5] Ali M Bahamondes L Bent Landoulsi S Extended effectiveness of the etonogestrel-releasing contraceptive implant and the 20 µg levonorgestrel-releasing intrauterine system for 2 years beyond U.S. Food and Drug Administration product labeling Glob Health Sci Pract 2017 5 04 534 539. Doi: 10.9745/GHSP-D-17-00296 29263025 10.9745/GHSP-D-17-00296 PMC5752601

[6] McNicholas C Swor E Wan L Peipert J F Prolonged use of the etonogestrel implant and levonorgestrel intrauterine device: 2 years beyond Food and Drug Administration-approved duration Am J Obstet Gynecol 2017 216 06 5860 5.86E8. Doi: 10.1016/j.ajog.2017.01.036 10.1016/j.ajog.2017.01.036 PMC5449254 28147241

[7] Bahamondes L Fernandes A Bahamondes M V Juliato C T Ali M Monteiro I Pregnancy outcomes associated with extended use of the 52-mg 20 μg/day levonorgestrel-releasing intrauterine system beyond 60 months: A chart review of 776 women in Brazil Contraception 2018 97 03 205 209. Doi: 10.1016/j.contraception.2017.10.007 29055780 10.1016/j.contraception.2017.10.007

[8] Xiao B L Zhou L Y Zhang X L Jia M C Luukkainen T Allonen H Pharmacokinetic and pharmacodynamic studies of levonorgestrel-releasing intrauterine device Contraception 1990 41 04 353 362. Doi: 10.1016/0010-7824(90)90035-t 2335100 10.1016/0010-7824(90)90035-t

[9] Natavio M F Taylor D Lewis R A Blumenthal P Felix J C Melamed A Temporal changes in cervical mucus after insertion of the levonorgestrel-releasing intrauterine system Contraception 2013 87 04 426 431. Doi: 10.1016/j.contraception.2012.09.034 23121828 10.1016/j.contraception.2012.09.034

[10] Moraes L G Marchi N M Pitoli A C Hidalgo M M Silveira C Modesto W Bahamondes L Assessment of the quality of cervical mucus among users of the levonorgestrel-releasing intrauterine system at different times of use Eur J Contracept Reprod Health Care 2016 21 04 318 322. Doi: 10.1080/13625187.2016.1193139 27269613 10.1080/13625187.2016.1193139

[11] Hidalgo M M Hidalgo-Regina C Bahamondes M V Monteiro I Petta C A Bahamondes L Serum levonorgestrel levels and endometrial thickness during extended use of the levonorgestrel-releasing intrauterine system Contraception 2009 80 01 84 89. Doi: 10.1016/j.contraception.2009.01.004 19501221 10.1016/j.contraception.2009.01.004

[12] Seeber B Ziehr S C Gschlieβer A Moser C Mattle V Seger C Quantitative levonorgestrel plasma level measurements in patients with regular and prolonged use of the levonorgestrel-releasing intrauterine system Contraception 2012 86 04 345 349. Doi: 10.1016/j.contraception.2012.01.015 22402256 10.1016/j.contraception.2012.01.015

[13] Teal S B Turok D K Chen B A Kimble T Olariu A I Creinin M D Five-year contraceptive efficacy and safety of a levonorgestrel 52-mg intrauterine system Obstet Gynecol 2019 133 01 63 70. Doi: 10.1097/AOG.0000000000003034 30531565 10.1097/AOG.0000000000003034 PMC6319579

[14] Apter D Gemzell-Danielsson K Hauck B Rosen K Zurth C Pharmacokinetics of two low-dose levonorgestrel-releasing intrauterine systems and effects on ovulation rate and cervical function: pooled analyses of phase II and III studies Fertil Steril 2014 101 06 1656 620, 4 24726226 10.1016/j.fertnstert.2014.03.004

[15] Goldstuck N D Clarification of the role of the Jaydess(Skyla) LNG- IUS 13.5mg and Kyleena LNG-IUS 19.5mg as intrauterine contraceptive systems Expert Rev Med Devices 2017 14 08 593 599. Doi: 10.1080/17434440.2017.1350169 28675069 10.1080/17434440.2017.1350169

[16] Gemzell-Danielsson K Inki P Boubli L O'Flynn M Kunz M Heikinheimo O Bleeding pattern and safety of consecutive use of the levonorgestrel-releasing intrauterine system (LNG-IUS)--a multicentre prospective study Hum Reprod 2010 25 02 354 359. Doi: 10.1093/humrep/dep426 19955104 10.1093/humrep/dep426

[17] Heikinheimo O Inki P Schmelter T Gemzell-Danielsson K Bleeding pattern and user satisfaction in second consecutive levonorgestrel-releasing intrauterine system users: results of a prospective 5-year study Hum Reprod 2014 29 06 1182 1188. Doi: 10.1093/humrep/deu063 24682613 10.1093/humrep/deu063 PMC4017944

[18] Belsey E M Machin D d'Arcangues C The analysis of vaginal bleeding patterns induced by fertility regulating methods. World Health Organization Special Programme of Research, Development and Research Training in Human Reproduction Contraception 1986 34 03 253 260. Doi: 10.1016/0010-7824(86)90006-5 3539509 10.1016/0010-7824(86)90006-5

[19] Rowe P Farley T Peregoudov A Piaggio G Boccard S Landoulsi S Safety and efficacy in parous women of a 52-mg levonorgestrel-medicated intrauterine device: a 7-year randomized comparative study with the TCu380A Contraception 2016 93 06 498 506. Doi: 10.1016/j.contraception.2016.02.024 26916172 10.1016/j.contraception.2016.02.024 PMC5357727

